# Once is rarely enough: can social prescribing facilitate adherence to non-clinical community and voluntary sector health services? Empirical evidence from Germany

**DOI:** 10.1186/s12889-020-09927-4

**Published:** 2020-11-30

**Authors:** Veronika Golubinski, Eva-Maria Wild, Vera Winter, Jonas Schreyögg

**Affiliations:** 1 Department of Health Care Management, Hamburg Center for Health Economics (HCHE), University of Hamburg, Esplanade 36, 20354 Hamburg, Germany; 2 Department of Health Care Management, Schumpeter School of Business and Economics, University of Wuppertal, Wuppertal, Germany

**Keywords:** Non-clinical health services, Community and voluntary sector, Germany, Social prescribing, Patient engagement, Deprivation

## Abstract

**Background:**

Non-clinical health interventions provided by the voluntary and community sector can improve patients’ health and well-being and reduce pressure on primary and secondary care, but only if patients adhere to them. This study provides novel insights into the impact of doctor referrals to such services, known as social prescribing, on patients’ adherence to them.

**Methods:**

Using a negative binomial model, we analysed electronic visitor records from a community health advice and navigation service in Germany between January 2018 and December 2019 to determine whether social prescribing was associated with greater adherence to the service (measured in terms of return visits) compared to patients who self-referred. We also explored whether this effect differed according to patient characteristics.

**Results:**

Based on 1734 observations, we found that social prescribing was significantly associated with a greater number of return visits compared to patient self-referrals (*p* < 0.05). For patients who visited the service because of psychological concerns, the effect of social prescribing was lower. For all other patient characteristics, the effect remained unchanged, suggesting relevance to all other patient groups.

**Conclusions:**

The results of our study indicate that social prescribing may be an effective way to facilitate adherence to non-clinical community and voluntary sector health services. This knowledge is important for policy makers who are deciding whether to implement or expand upon social prescribing schemes.

**Supplementary Information:**

The online version contains supplementary material available at 10.1186/s12889-020-09927-4.

## Background

Patients often do not differentiate between social and medical problems when seeking help from general practitioners (GPs) or other health professionals. Particularly in socially deprived areas, many patients present with concerns that arise from their personal and social circumstances [1–3]. However, while such circumstances often affect the management of long-term medical conditions [3], the complex social, mental and physical problems they entail often cannot be dealt with effectively by GPs alone and require community and voluntary sector resources [4, 5].

Non-clinical health services provided by community or voluntary sector organisations can help relieve GPs’ workload, reduce inequalities in health care and improve patients’ health and quality of life [6–11]. Indeed, when medical treatments reach their limits or prove ineffective for some patients, GPs consider sources of support in the community to be particularly helpful [12]. Many countries have therefore introduced schemes that enable primary care professionals to refer patients to such services [13], a practice known variously as social prescribing, non-medical referral or community referral [14, 15]. Examples of support include management of mental health, advice on weight and long-term conditions, art therapy, learning/employment assistance and exercise classes [11].

To date, research on social prescribing schemes has focused primarily on the English NHS and yielded mixed results regarding their effectiveness and efficiency in improving patient outcomes and reducing GP workload [10, 16–18]. However, despite this inconclusive evidence, there is a consensus that any positive effects of referring patients to sources of support in the community can only be expected if patients show ongoing involvement with (i.e., adhere to) them [15, 19]. Indeed, studies have reported that the likelihood of achieving positive health-related outcomes increases with the frequency with which a patient uses such services [20–23]. Additionally, research also suggests that social prescribing probably has a positive effect on the use of community and voluntary sector services [15]. Moreover, the findings of a number of qualitative studies suggest that patients’ relationship to their GP facilitates adherence to GPs’ recommendations [24–28]. However, quantitative evidence on whether doctor referral (i.e., social prescription) facilitates adherence to non-clinical health services in the community is lacking.

To address this gap in the literature, we compared two distinct patient pathways to accessing a community health advice and navigation service in Germany: social prescription vs. self-referral. We took a process evaluation perspective and investigated whether patients with a social prescription were more likely adhere to services than were patients who self-refer. Second, we investigated whether the two patient pathways differed in their effect on adherence depending on patients’ characteristics. Knowing whether social prescribing can promote patient adherence and whether it might be more effective for certain groups of patients is important for policy makers who are deciding whether to implement or expand upon such schemes.

## Methods

### Research setting and data

Like the health care systems in countries such as the United Kingdom, Ireland and the Netherlands, the German health care system is also exploring ways to enable health professionals to refer patients to local, non-medical health services provided by community and voluntary sector organisations. Our analyses use data from the first such service in Germany to which GPs and specialists can refer patients by means of a social prescription. The community health advice and navigation service opened on 26 September 2017 and is part of a patient-oriented and cross-sectoral healthcare network aiming to improve healthcare in a district of northern Germany. The district is home to a multi-cultural population of approximately 110,000 people and has a moderate level of deprivation. The organization through which the community health interventions are provided is a privately owned regional management company (German entrepreneurial company with limited liability). It is not run as a registered charity. The management company received 3 years of start-up funding from the Innovation Fund of the German Federal Joint Committee (G-BA) and is now financed by several statutory health insurers. While the legal form is a for-profit company, the company has a non-profit distributing mission and it’s financing through the statutory health insurers speaks for considering it part of the voluntary and community sector, as are community service providers in other settings and countries. The service in our study can be understood as a one-stop-shop for health-related information, disease prevention, and health promotion. It offers one-to-one sessions with nurses and allied health workers, where patients can receive health advice and education (e.g., dietary change, smoking cessation, social and family issues, preparation of administrative procedures) in their mother language to better understand their individual burden of disease and opportunities for prevention. The nurses and allied health workers at the service cover eight of the most spoken languages in the neighbourhood. While this type of service is highly tailored and personalised to individual needs, the community health advice and navigation service in our study also offers group interventions. These group interventions have a more generic orientation and address specific patient groups (e.g. obese patients or patients with mental health problems). Both types of services are provided at the premises of the health advice and navigation service. In addition, similar to social prescribing in other countries such as the UK, the health care navigation and advice service involves and cooperates with existing local community services/groups/activities. Specifically, the service informs patients about the local community services and supports patients in finding and arranging appointments with the appropriate health professionals, welfare institutions, exercise groups and wellbeing groups. To determine which types of service an individual needs, nurses and allied health workers who are employed at the community health advice and navigation service undertake a comprehensive needs assessment and build-up a tailored care plan for the patient during their first consultation. For patients with a referral, the information on diagnosis or suspected disease and treatment recommendation/plan provided by the GP/specialist on the referral form is considered in this step. While there is no formal link worker as in other settings, like the UK, the nurses and allied health workers who are employed at the community health advice and navigation service partly take over the link worker functions, such as patients’ need assessment and coordinating with existing local community services/groups/activities. The service is free at point of use for all patients, regardless of whether they have a social prescription or self-refer.

The fact that patients can use the service either by obtaining a social prescription from a doctor or by self-referring allowed us to estimate directly the impact of social prescribing on patients’ adherence to the service. In this context, it should be noted that GPs in Germany do not function as gatekeepers to secondary care, the majority of which is provided in office-based practices and can be accessed by patients directly without a referral. Both GPs and consultants/specialist doctors were able to refer patients to the health care navigation and advice service examined in this study.

Data collection started on 1 January 2018. To measure service utilization, we created a cross-sectional data set by merging several sources of data maintained by the health advice and navigation service and using an ID for each of its visitors. The data set accounts for all patients who visited the service at least once from 1 January 2018 to 31 December 2019. To ensure comparability, we excluded patients younger than 18 years from our analyses (*n* = 110), yielding a final set of data from 1734 patients who visited the service at least once in the observation period. The study was approved by the Institutional Review Board of the University of Hamburg.

### Measures

Table 1 gives a description of the study’s variables*.*

**Table 1:** Description of study variables

Variable	Description
Number of return visits	Number of times a patient visited the health advice and navigation service within 3 months after the initial visit Count variable
Social prescription	Binary variable 0 = Patient self-refers to the service 1 = Patient is referred to the service by a doctor
Gender	Binary variable 0 = Female 1 = Male
Age	Age in years (continuous variable)
Distance	Geographical distance in km from the patient’s residence to the service (continuous variable)
Visit due to overweight	Binary variable 0 = Reason for visiting the service was not overweight or obesity 1 = Reason for visiting the service was overweight or obesity
Visit due to psychological concern	0 = Reason for visiting the service was not a psychological concern 1 = Reason for visiting the service was a psychological concern

#### Dependent variable

We used the number of times patients attended the health advice and navigation service during the 3 months following their initial visit (henceforth described as the number of return visits) as a proxy for patient adherence to it within the observation period. A similar understanding of patients’ adherence to such services has been used by other authors in the field of social prescribing, including Pescheny et al. [24] and Husk et al. [15]. As our observation period, we used the mean time between patients’ initial and last visit in our sample, which was 3 months. Because there is no common definition of, or empirical evidence on, the optimal length of an observation period for counting return visits, we conducted sensitivity analyses using observation periods of different lengths to test the robustness of our results (see sensitivity analyses in the results section).

#### Independent variables

The main variable of interest was “social prescription”, which was a binary variable and equal to one if a patient was referred to the service by a primary or secondary care professional or zero if the patient had self-referred to the service.

We controlled for variables suggested in prior research to be relevant to patient engagement and adherence, namely gender [21, 29–31], age [21, 32, 33], geographical distance to the service [15] and type of illness or reason for using the service, i.e. overweight or obesity or a psychological concern [34, 35].

### Statistical analyses

Because our dependent variable (i.e., the number of return visits) is count data, we compared the fit of four models: the simple Poisson regression model, the zero-inflated Poisson model, the simple negative binomial model and the zero-inflated negative binomial model. First, we used a likelihood ratio test and assessed the over-dispersion parameter α to investigate whether a negative binomial model would be more appropriate for our analysis than a Poisson model. If α is significantly greater than zero, then the data are over-dispersed and are better estimated using the former [36]. Indeed, this was the case in our dataset (α = 0.718, *p* < 0.01). Second, we compared model residuals and different model fit statistics to assess whether a zero-inflated negative binomial model would provide more precise estimates. For this purpose, we used a graphical comparison of model residuals (see Figure A), a chi^2^ test to compare the actual distribution of the data and the distribution proposed by the models, and the Bayesian Information Criterion (BIC). The results of all tests suggested that the negative binomial model was the most appropriate for our analysis. Lastly, a chi^2^ goodness-of-fit test showed that there was generally no evidence of misspecification in our negative binomial model. We provide further information on the model fit statistics in Additional file 1: Appendix A. To investigate whether the social prescription effect differed depending on patient characteristics, we also tested interaction effects of the variable social prescription with the control variables.

To facilitate interpretation of our results, we transformed coefficients into incidence rate ratios (IRR). The IRR is defined as e^β^ and represents the average number of return visits to the health advice and navigation service with respect to a one-unit increase in the explanatory variables used in this study. In anticipation that we might have misspecified the true (but unknown) population density, we chose robust standard error procedures.

### Sensitivity analyses

To test the robustness of our results, we performed two sets of sensitivity analyses.

First, we tested the robustness of our results using periods of one, two, four, five and 6 months.

Second, patients referred to the health advice and navigation service by a doctor might have had more complex needs, and therefore made a larger number of return visits, than patients without such a referral. To rule out this form of selection bias, we conducted a subsample analysis using data from the 197 patients for whom we had health status data and included several health-related variables, i.e. body mass index, sports activity, smoking, diabetes, hypertension and back pain. We used two-sample t-tests for continuous variables and chi^2^-tests for binary variables to compare the two groups: patients with a social prescription and patients who self-referred.

We performed all analyses with STATA version 15.1 (College Station, TX).

## Results

### Descriptive statistics

Table 2 summarizes the individual-level descriptive statistics for patients with a social prescription (36% of the sample) and those who self-referred (64%). The gender distribution, mean age and average distance from patients’ homes to the service did not differ significantly between the two groups. The percentage of those who visited the service due to overweight differed significantly between the two groups, 48% of whom had a social prescription and 33% of whom had self-referred. In both groups, 5% of patients visited the service because of a psychological concern.

**Table 2:** Summary statistics

*N* = 1734	With social prescription (*N* = 622)				Self-referral (*N* = 1112)				Difference
Variable	Mean	SD	Min	Max	Mean	SD	Min	Max	
Male (in %)	0.30	0.46	0	1	0.30	0.46	0	1	0
Age (in years)	57.06	14.78	18	98	57.21	16.06	18	96	0.15
Distance (in km)	3.33	3.50	0.03	23.89	3.37	3.43	0.17	23.57	0.04
Visit due to overweight (in %)	0.48	0.50	0	1	0.33	0.47	0	1	0.15***
Visit due to psychological concern (in %)	0.05	0.22	0	1	0.05	0.22	0	1	0

This table presents the summary statistics, grouped by patients with social prescription (left part) and without social prescription, i.e. who self-referred to the service (right part). Two-sample t-tests for continuous variables and chi^2^-tests for binary variables were used to compare the two groups

All the *p*-values have been replaced by stars and categorised as follows. ***: *p* < 0.01; **: *p* < 0.05; *: *p* < 0.10

### Regression results

The results of our regression are summarized in Table 3. The first column contains the IRRs for a model without control variables; the second column contains the results of the full model including the control variables. The incidence rate of return visits for patients who had a social prescription was 20% higher (IRR: 1.204) than that for patients who did not (*p* < 0.01). The effect remained stable (IRR 1.166, *p* < 0.05) after the inclusion of control variables.

**Table 3:** Main results: Effect of a social prescription on number of return visits

	*IRR for number of return visits*	
*Variable*	*(1)*	*(2)*
Social prescription (=1)	1.204*** (0.076)	1.166** (0.074)
Male (=1)		0.897 (0.064)
Age (in years)		0.997 (0.002)
Distance (in km)		0.978** (0.008)
Visited due to overweight		1.251*** (0.082)
Visit due to psychological concern (=1)		1.003 (0.173)
Observations	1734	1734

Negative binomial models are estimated. IRR is the incidence rate ratio defined as e^β^, where β is the coefficient estimate. Robust standard errors are presented in parentheses

All the *p*-values have been replaced by stars and categorised as follows. ***: *p* < 0.01; **: *p* < 0.05; *: *p* < 0.10

Regarding the control variables, neither gender nor age significantly affected the number of return visits. The closer patients lived to the service, the higher the number of their return visits. Finally, visiting the service due to overweight or obesity significantly increased the number of return visits.

Table 4 summarizes the results of our interaction analyses, which investigated whether a social prescription might be more effective for certain groups of patients. We found that the interactions between having a social prescription and patients’ characteristics with respect to their gender, age and visiting the service due to overweight were not statistically significant at conventional significance levels. This indicates that the effect of a social prescription did not vary depending on these patient characteristics, thus suggesting effectiveness to all patient groups. At the same time, however, we found a statistically significant negative interaction effect for having visited the service because of psychological concerns, suggesting that the effect of a social prescription on the number of return visits was lower for these patients compared to those who did not visit the service for this reason. To get a better impression of the effect’s size for each group, we provide a graphical illustration in Additional file 2: Appendix B (see Figure B).

**Table 4:** Effect of a social prescription on number of return visits including interactions

*Dependent variable = Number of return visits*	(1)	(2)	(3)	(4)	(5)
Social prescription (=1)	1.162** (0.086)	0.807 (0.192)	1.149 (0.100)	1.204* (0.114)	1.201*** (0.079)
Male (=1)	0.893 (0.080)	0.891 (0.063)	0.898 (0.063)	0.896 (0.064)	0.893 (0.063)
Age (in years)	0.997 (0.002)	0.995* (0.003)	0.997 (0.002)	0.997 (0.002)	0.997 (0.002)
Distance (in km)	0.978*** (0.009)	0.977*** (0.009)	0.976** (0.011)	0.978*** (0.009)	0.978** (0.009)
Visit due to overweight (=1)	1.257*** (0.082)	1.257*** (0.082)	1.256*** (0.082)	1.292*** (0.107)	1.250*** (0.082)
Visit due to psychological concern (=1)	1.056 (0.183)	1.059 (0.183)	1.055 (0.183)	1.056 (0.184)	1.294 (0.265)
Social prescription × Male	1.013 (0.148)				
Social prescription × Age		1.006 (0.004)			
Social prescription × Distance			1.005 (0.018)		
Social prescription × Visit due to overweight				0.932 (0.118)	
Social prescription × Visit due to psychological concern					0.519** (0.169)
Observations	1734	1734	1734	1734	1734

This table presents mean interaction effects and robust standard errors (in parentheses) of the negative binomial model. The dependent variable is *Number of Returns.* The main variable of interest is *Social Prescription*. The incidence rate ratio (IRR) is reported. All the *p*-values have been replaced by stars and categorised as follows. ***: *p* < 0.01; **: *p* < 0.05; *: *p* < 0.10

### Sensitivity analyses

The results of the first set of sensitivity analyses using observation periods of lengths other than 3 months are presented in Additional file 3: Appendix C. The results suggest that our main effect of interest remained stable. The only exceptions were the coefficients for the five- and six-month observation periods, which were no longer significant when we included control variables in our regressions (see columns 10 and 12). This may be attributable to the decrease in the size of the sample as the length of the observation period increases.

For the second sensitivity analysis, our results suggest that health status did not significantly differ between the patients with or without a social prescription, indicating that selection bias due to health status did not substantially influence our results (see Additional file 4: Appendix D).

## Discussion

Our work provides the novel insight that social prescriptions can have a powerful impact on whether patients adhere to non-clinical health services provided by the voluntary and community sector. In particular, we found that patients who had a social prescription had better adherence to a community-sector health advice and navigation service in Germany compared to patients who self-referred.

Our study expands upon previous research by explicitly comparing two distinct pathways that patients can take to use such services: social prescription versus self-referral. Understanding these pathways is essential to identifying the ways in which social prescriptions might be of greatest benefit and to designing effective social prescribing schemes [14, 15, 19].

In addition, our study adds important insights to the mixed evidence available on the effectiveness and efficiency of such schemes in reducing GP workload and healthcare demand more generally [16, 17, 37, 38]. In particular, the non-significant findings of many previous studies might be partially explained by the multicausal nature of their outcome indicators. By using a more proximal indicator, we provide evidence that social prescribing may indeed be beneficial in stimulating adherence to community and voluntary sector services. Investigating process indicators offers important insights into how social prescribing works and, in turn, enhances our understanding of the mechanisms through which outcomes can be achieved [39]. Further research on social prescribing schemes could analyse other process indicators that, like adherence, are more proximate to the receipt of a social prescription and are expected to bring about change in more distal, but ultimately more meaningful indicators such as health and quality of life.

Qualitative studies in this area of enquiry may help explain the mechanisms behind the effect of social prescribing we observed in our quantitative analysis. In particular, trust in GPs [24, 27] and respect for a GPs’ authority [40, 41] have been found to increase patients’ adherence to social prescriptions. Patients with social prescriptions might be more confident in the necessity of service than patients who self-refer. Indeed, GPs may also function as a catalyst for behavioural change and have a “priming effect” on patients’ responses to health-related interventions [42].

Our interaction analyses suggest that patients who visit a community-based service because of psychological concerns are not more likely to adhere to services if they have a social prescription as opposed to self-referral. This finding is corroborated by previous research, which has found that patients with mental illnesses are less likely to adhere to referral schemes [27, 32] or to complete referral programmes [43]. While the explanations for this are numerous, one important reason for non-compliance with social prescriptions appears to be a self-perceived lack of need for continuing treatment [44, 45]. At a more practical level, our findings from the interaction analysis suggest that health care practitioners should target subgroups of patients for whom social prescribing is especially effective, i.e., patients who do not have a psychological concern. In contrast, patients with a psychological concern might need additional sources of support such as mentoring programmes or link workers to improve their adherence to non-clinical community and voluntary sector health services. A link worker could emphasize the personal need and benefits of continuously using the non-clinical community and voluntary sector health services. Moreover, further research on social prescribing could analyse other individual-level characteristics that, like psychological concerns, might moderate the effect of a social prescription on adherence. This might be helpful to better understand how policy makers and health care decision makers can maximize the effectiveness of social prescriptions.

Our study has several important limitations, each of which offers opportunities for further research. First, our results may be subject to omitted variable bias because our dataset did not allow us to include individual patient characteristics at a more granular level. In particular, psychosocial factors (e.g. self-efficacy, patient activation, social support) as well as health-related factors may have directly or indirectly influenced the effect of social prescriptions. Although the results of our sensitivity analyses (see Additional file 4: Appendix D) suggest that bias due to health-related factors does not substantially affect our results, we nevertheless encourage future researchers to replicate and expand upon our analyses by using a richer set of individual characteristics to identify more precisely which patients are most likely to benefit from social prescribing. In addition, ease of access might affect our results [13]. In our study, we used geographical distance as proxy for “ease of access”. The distance to the service did not differ significantly between the group of patients with social prescription and the group of patients who self-refer. However, controlling for geographical distance in our regression model, we found a significant association between geographical distance and patients’ adherence. Considering that access is a multi-dimensional concept, we encourage future research to account for other aspects and barriers that might limit the utilisation of services, such as opening hours or costs of using the service.

A second limitation of our study is its focus only on one community sector provider, which might oversimplify the phenomenon of social prescribing. In other countries, such as the UK, social prescribing schemes include prescriptions to a broad range of community and voluntary sector services. Indeed, Husk et al. [13] point out that social prescribing should not be seen as a single intervention, but rather a series of relationships between referrer, patient, link worker and activity, all of whom interact with one other. The absence of a link worker sets the social prescribing intervention in our study apart from other social prescribing schemes (e.g., in the UK) and thus limits the generalisability of our findings beyond our study setting. However, this setting allows us to isolate the social prescription ‘referral’ effect from third party influence. A link worker will likely co-influence the effect of a social prescription (i.e. referral from GP to health advice and navigation service) on patients’ adherence to services. Further our focus on one service has the advantage that we could rule out the presence of unobservable social prescribing activities in our analysis. Further research could replicate our analyses in social prescribing settings that encompass link workers as well as multiple services and activities. This will allow to quantify the additional benefits of a link worker to increase patients’ adherence. Also, it would be interesting to examine whether the social prescribing effects differ across health systems (e.g., in more integrated health systems, such as the English NHS).

While not a limitation, another point for future research is related to the setting of our study, which was conducted in a socially deprived urban area. Such areas are the setting for which social prescribing was originally conceived and in which there is a strong need to address the social determinants of health and wellbeing in addition to providing medical care [14, 46]. However, Moore et al. [27] showed that GPs faced greater difficulties in deprived areas when attempt to connect patients with non-clinical community and voluntary sector health services (in this case, an exercise programme) through social prescribing. Other research from a different context (i.e., doctor referrals to specialty care) has found that living in a deprived urban area is negatively associated with adherence to doctor referrals [47]. Bearing this in mind and assuming that, in the absence of a social prescription, adherence in urban areas that are deprived does not differ from that in urban areas that are wealthier, our main effect may even represent an underestimate. Put differently, the effect of a social prescription on adherence might be higher in wealthier urban areas. We leave this conjecture, however, to be investigated in future work.

## Conclusion

Non-clinical health services provided by the community or voluntary sector are important as they can help reduce the burden on primary and secondary care and improve patients’ quality of life. The findings of our study suggest that social prescriptions are a promising tool to increase adherence to these services and may therefore be useful to policy makers deciding whether to implement or expand upon social prescribing schemes. Our finding that this effect did not differ according to patients’ age, gender or geographic proximity suggests that social prescriptions may be a broadly applicable intervention with cross-community benefits.

## Supplementary Information

**Additional file 1 **: **Appendix A** [48, 49] provides further information on model fit statistics as a means to compare the zero-inflated negative binomial model to a traditional negative binomial model. **Figure A**: Graphical illustration of the residuals from the negative binomial model and zero-inflated negative binomial model. **Figure A** illustrates the residuals from the negative binomial model and zero-inflated negative binomial model.

**Additional file 2 **: **Appendix B** provides additional information on our interactions analyses. **Figure B**: Interaction effect between social prescription and visit due to psychological concerns on return visits. **Figure B**: illustrated the interaction effect between social prescription and visit due to psychological concerns on return visits.

**Additional file 3 **: **Appendix C** shows the results of our sensitivity analyses using observation periods of lengths other than 3 months.

**Additional file 4 **: **Appendix D** shows the results of our subgroup analysis on health-related characteristics.

## Data Availability

The data that support the findings of this study are available from *Gesundheit für Billstedt/Horn UG* but restrictions apply to the availability of these data, which were used under license for the current study, and so are not publicly available. Data are however available from the authors upon reasonable request and with permission of *Gesundheit für Billstedt/Horn UG*.

## Abbreviations

α: Over-dispersion parameter α
BIC: Bayesian Information Criterion
GP: General practitioner
ID: Identifier
IRR: Incidence Rate Ratio
NBRM: Negative binomial model
SD: Standard deviation
UK: United Kingdom
ZINB: Zero-inflated negative binomial model
