# Supplementary material for: Once is rarely enough: can social prescribing facilitate adherence to non-clinical community and voluntary sector health services? Empirical evidence from Germany
Source: BMC Public Health. 2020 Nov 30;20:1827. doi: 10.1186/s12889-020-09927-4 (PMC7706247; doi:10.1186/s12889-020-09927-4)
Supplement: Supplementary file 1 — Additional file 1 : Appendix A [48, 49] provides further information on model fit statistics as a means to compare the zero-inflated negative binomial model to a traditional negative binomial model. Figure A: Graphical illustration of the residuals from the negative binomial model and zero-inflated negative binomial model. Figure A illustrates the residuals from the negative binomial model and zero-inflated negative binomial model. [file 12889_2020_9927_MOESM1_ESM.docx]

**Appendix A**

This appendix provides further information on model fit statistics as a means to compare the zero-inflated negative binomial model to a traditional negative binomial model.

*Graphical comparison of model residuals*

The graph in figure A plots the residuals from the negative binomial model (NBRM) and zero-inflated negative binomial model (ZINB). Small residuals are an indicator of good-fitting models. Hence, the model with a line closer to zero should be considered for our data. We see in the graph that the negative binomial model and zero-inflated negative binomial model perform very similarly for counts greater than two, and that they both differ most from the actual values and from each other at the zero, one and two counts. At the zero, one and two counts, the negative binomial model appears slightly better than the zero-inflated negative binomial model.

*
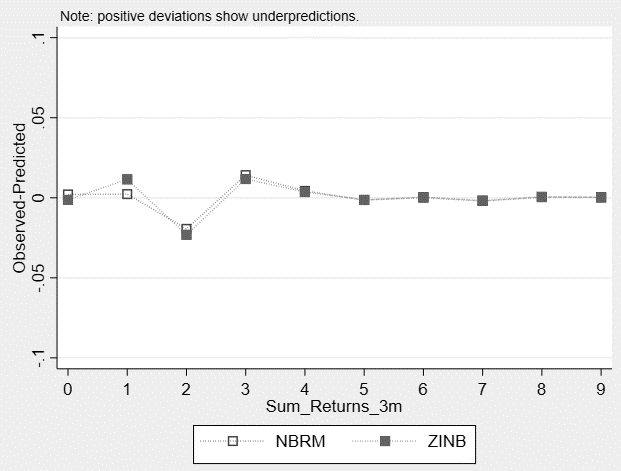
*

*Comparison of model residuals by count*

Looking at the maximum and mean differences in observed versus predicted counts, we see that both models performed worst when predicting a count of two. We observe that the negative binomial performs better at the prediction of the count of two and, overall, has a lower mean difference (0.005 vs. 0.006) between predicted and observed values. This confirms what we observed in the graph above.

Also when using a Pearson chi² test to compare the actual distribution of the data and the distribution proposed by the two models, the negative binomial model seems to perform better than the zero-inflated negative binomial (negative binomial model: sum of Pearson= 9.835; zero-inflated negative binomial model: sum of Pearson= 10.187).

*Bayesian Information Criterion*

The Bayesian Information Criterion (BIC) indicates that the negative binomial model outperforms the zero-inflated negative binomial model (negative binomial model: BIC= 3057.763; Zero-inflated negative binomial model: BIC= 3078.602).

*Chi² goodness-of-fit test*

Following Manjón et al. [48], we use the chi² goodness-of-fit test developed by Andrews et al. [49]. In applications, a model might be misspecified (that is, the model moment conditions are not satisfied) if the chi² goodness-of-fit test is statistically significant at conventional levels. Otherwise, there is no evidence of model misspecification, which was the case for our negative binomial model.
