# Supplementary material for: Once is rarely enough: can social prescribing facilitate adherence to non-clinical community and voluntary sector health services? Empirical evidence from Germany
Source: BMC Public Health. 2020 Nov 30;20:1827. doi: 10.1186/s12889-020-09927-4 (PMC7706247; doi:10.1186/s12889-020-09927-4)

**Appendix B**

This appendix provides additional information on our analyses resulting from interactions. In addition to our regression results from the main text, we provide a margins plot from the “Social Prescription x Visit due to psychological concerns” interaction that was found to be significant. Figure B shows that a social prescription had a positive effect on the number of return visits for patients who were not visiting the service because of psychological concerns (see left side of the figure). For this group of patients, the average number of return visits was significantly higher among those who had a social prescription compared to those who self-referred. For patients who visited the service because of psychological concerns, the effect of a social prescription on the average number of return visits was indistinguishable from zero (as displayed by the confidence interval).


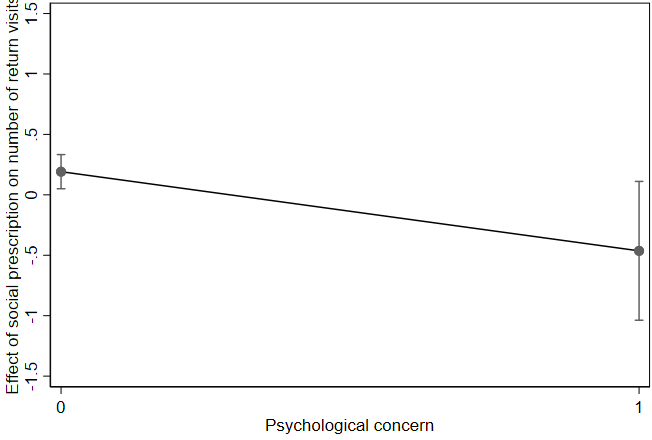

Supplement: Supplementary file 2 — Additional file 2 : Appendix B provides additional information on our interactions analyses. Figure B: Interaction effect between social prescription and visit due to psychological concerns on return visits. Figure B: illustrated the interaction effect between social prescription and visit due to psychological concerns on return visits. [file 12889_2020_9927_MOESM2_ESM.docx]
