# Supplementary material for: Once is rarely enough: can social prescribing facilitate adherence to non-clinical community and voluntary sector health services? Empirical evidence from Germany
Source: BMC Public Health. 2020 Nov 30;20:1827. doi: 10.1186/s12889-020-09927-4 (PMC7706247; doi:10.1186/s12889-020-09927-4)
Supplement: Supplementary file 3 — Additional file 3 : Appendix C shows the results of our sensitivity analyses using observation periods of lengths other than 3 months. [file 12889_2020_9927_MOESM3_ESM.docx]

**Appendix C**

**Table C**
The effect of social prescription for observation periods of different lengths

|  | *Return visits  over one month* | |  | *Return visits  over two months* | |  |  | *Return visits over four months* | |  | *Return visits  over five months* | |  | *Return visits  over six months* | |
| --- | --- | --- | --- | --- | --- | --- | --- | --- | --- | --- | --- | --- | --- | --- | --- |
| *Variable* | *(1)* | *(2)* |  | *(3)* | *(4)* |  |  | *(7)* | *(8)* |  | *(9)* | *(10)* |  | *(11)* | *(12)* |
| Social prescription (=1) | 1.176** (0.075) | 1.147** (0.074) |  | 1.198*** (0.074) | 1.169** (0.073) |  |  | 1.142** (0. 076) | 1.101 (0. 074) |  | 1.143* (0.081) | 1.102 (0.078) |  | 1.149* (0.087) | 1.111 (0.084) |
| Male (=1) |  | 0.982 (0.069) |  |  | 0.954 (0.066) |  |  |  | 0.925 (0.069) |  |  | 0.893 (0.070) |  |  | 0.878 (0.076) |
| Age (in years) |  | 0.998 (0.002) |  |  | 0.996** (0.002) |  |  |  | 0.998 (0.002) |  |  | 1.000 (0.002) |  |  | 0.999 (0.003) |
| Distance (in km) |  | 0.985* (0.008) |  |  | 0.983* (0.009) |  |  |  | 0.978** (0.009) |  |  | 0.980** (0.010) |  |  | 0.979* (0.010) |
| Visit due to overweight (=1) |  | 1.224*** (0.081) |  |  | 1.229*** (0.078) |  |  |  | 1.331*** (0.091) |  |  | 1.317*** (0.096) |  |  | 1.291*** ( 0.101) |
| Visit due to psychological concerns (=1) |  | 1.089 (0.179) |  |  | 1.067** (0.171) |  |  |  | 1.198 (0.222) |  |  | 1.187 (0. 228) |  |  | 1.129 (0.235) |
| Observations | 1,948 | 1,948 |  | 1,832 | 1,832 |  |  | 1,625 | 1,625 |  | 1,534 | 1,534 |  | 1,435 | 1,435 |

Note: All regressions are negative binomial models. Robust standard errors are presented in parentheses. The incidence rate ratio (IRR) is reported. Sample sizes differ for different time periods. The longer the chosen time period, the smaller the sample gets. Statistical significance levels: *p < 0.10, **p < 0.05, ***p < 0.01
