# Supplementary material for: Once is rarely enough: can social prescribing facilitate adherence to non-clinical community and voluntary sector health services? Empirical evidence from Germany
Source: BMC Public Health. 2020 Nov 30;20:1827. doi: 10.1186/s12889-020-09927-4 (PMC7706247; doi:10.1186/s12889-020-09927-4)
Supplement: Supplementary file 4 — Additional file 4 : Appendix D shows the results of our subgroup analysis on health-related characteristics. [file 12889_2020_9927_MOESM4_ESM.docx]

**Appendix D**

**Table D**
Subgroup analysis on health-related characteristics

| N=197 | Mean | | Difference |
| --- | --- | --- | --- |
| Variable | With social prescription | Self-referral |  |
| Body Mass Index | 31.462 | 31.063 | 0.399 |
| Sports activity (=1) | 0.376 | 0.268 | 0.108 |
| Smoking (=1) | 0.224 | 0.232 | 0.008 |
| Diabetes (=1) | 0.224 | 0.205 | 0.019 |
| Hypertension (=1) | 0.259 | 0.277 | 0.018 |
| Back pain (=1) | 0.165 | 0.170 | 0.005 |
| Observations | 85 | 112 |  |

Note: This table presents the results of an additional analysis with a subsample of 197 observations. A two-sample t-test for the continuous variable (Body Mass Index) and chi²-test for binary variables (sports activity, smoking, diabetes, hypertension, back pain) was used to compare patients with social prescription (left part) and without social prescription, i.e. who self-referred to the service (right part). Results show that health-related characteristics do not significantly differ in both groups. Statistical significance levels: *p < 0.10, **p < 0.05, ***p < 0.01
